# Metabolic syndrome may be associated with a lower prevalence of iron deficiency in Ecuadorian women of reproductive age

**DOI:** 10.1017/jns.2020.55

**Published:** 2021-01-12

**Authors:** Melisa A. Muñoz-Ruiz, Laura I. González-Zapata, Victoria Abril-Ulloa, Diego A. Gaitán-Charry

**Affiliations:** 1 Unidad de Problemáticas de Interés en Nutrición Pública, Escuela de Nutrición y Dietética, Universidad de Antioquia UdeA, Calle 70 No. 52-21, Medellín, Colombia; 2 Grupo de investigación Determinantes Sociales y Económicos de la Situación de Salud y Nutrición, Escuela de Nutrición y Dietética, Universidad de Antioquia UdeA, Calle 70 No. 52-21, Medellín, Colombia; 3 Grupo de investigación Salud Pública, Alimentación y Actividad física en el ciclo de la vida, Carrera de Nutrición y Dietética, Facultad de Ciencias Médicas, Universidad de Cuenca, Cuenca, Ecuador; 4 Dirección de Investigación de la Universidad de Cuenca, Cuenca, Ecuador

**Keywords:** Metabolic syndrome, Iron deficiency, Iron-deficiency anaemia, Women

## Abstract

The present study aimed to assess the associations of the stages of Fe deficiency (Fe deficiency without anaemia (ID) and Fe-deficiency anaemia (IDA)) and anaemia with metabolic syndrome (MetS) in Ecuadorian women. A cross-sectional study was conducted in 5894 women aged 20–59 years, based on data from the 2012 Ecuadorian National Health and Nutrition Survey. The sample was stratified by age. A *χ*^2^ test was used to assess the possible associations of ID, IDA and anaemia with MetS. The prevalence ratio (PR) for each stage of Fe deficiency and anaemia was estimated considering women without MetS as a reference. The total prevalence of MetS, ID, IDA and anaemia was 32⋅3 % (se 0⋅6), 6⋅2 % (se 0⋅3), 7⋅1 % (se 0⋅3) and 5⋅0 % (se 0⋅3), respectively. In women aged 20–29, 30–39 and 40–49 years, MetS was associated with a lower prevalence of ID (PR (95 % CI; *P*-value)): 0⋅17 (0⋅06, 0⋅46; *P* < 0⋅001), 0⋅69 (0⋅48, 0⋅99; *P* = 0⋅044) and 0⋅44 (0⋅29, 0⋅67; *P* < 0⋅001), respectively. In women aged 50–59 years, MetS was associated with IDA and anaemia (PR (95 % CI; *P*-value)): 0⋅12 (0⋅02, 0⋅96; *P* = 0⋅026) and 0⋅22 (0⋅07, 0⋅64; *P* = 0⋅002), respectively. In conclusion, Ecuadorian women of reproductive age with MetS have a lower prevalence of ID compared with those without MetS. Furthermore, the MetS and IDA coexist at the population level. These findings require an analysis from a dietary pattern approach, which could provide key elements for developing public policies that simultaneously address all forms of malnutrition.

## Introduction

The double burden of malnutrition (DBM) is characterised by the coexistence of undernutrition (or micronutrient deficiency) and overweight or diet-related non-communicable diseases (NCDs)^(1)^. DBM is associated with a change in the dietary patterns of the population from a natural diet to a diet with a high content of ultra-processed products^(2,3)^. It is estimated that more than 20 % of Latin American women have metabolic syndrome (MetS)^(4–7)^, whereas the prevalence of anaemia is 22 % (in women aged 14–49 years)^(8)^. MetS increases the risk of cardiovascular diseases and diabetes^(9)^, which are the leading causes of death worldwide^(10)^. On the other hand, anaemia and Fe-deficiency anaemia (IDA) affect physical performance, work productivity^(11)^ and health outcomes during pregnancy and birth^(12)^. Furthermore, in non-anaemic women, Fe deficiency is associated with anger and fatigue^(13)^, and reduced body Fe is associated with decreased performance on cognitive executive planning function^(14)^. Even though an individual may be affected by both sides of DBM^(1)^, the association between these sides is still unclear.

Several studies have evaluated the association between overweight and Fe status. Analysis from the Mexican National Nutrition Survey 1999 showed that, in women (18–50 years), obesity is associated with Fe deficiency (OR = 1⋅92; 95 % CI 1⋅23, 3⋅01; *P* < 0⋅05). Although Fe intake in both obese and non-obese was similar, obese had a lower serum Fe concentration than non-obese (mean (sd) = 62⋅6 (29⋅5) μg/dl *v.* 72⋅4 (34⋅6) μg/dl; *P* = 0⋅014)^(15)^. Moreover, a recent study including school-aged children from Guangzhou, China, found that obesity was associated with a lower risk of anaemia (adjusted OR = 0⋅553; 95 % CI 0⋅316, 0⋅968; *P* = 0⋅038) and a higher risk of Fe deficiency without anaemia (ID) (adjusted OR = 1⋅808; 95 % CI 1⋅146, 2⋅853; *P* = 0⋅011). However, in this work, obesity was not associated with IDA^(16)^. The underlying mechanisms for the above-mentioned associations remain unclear. It is postulated that obesity-related inflammation induces overproduction of hepcidin, a key modulator of Fe metabolism, which affects Fe status by decreasing its absorption and increasing intracellular sequestration^(17)^. Nonetheless, other authors have not found the association of obesity with Fe deficiency without anaemia in Mexican women^(18)^ or with anaemia in Colombian women^(19)^.

Considering the latter discrepancy, it is interesting to evaluate the association between MetS and Fe status. A meta-analysis of cross-sectional and prospective studies, comprising data from 78 851 subjects, reported a positive association of high serum ferritin levels (a biomarker of Fe stores) with MetS (OR = 1⋅78; 95 % CI 1⋅60, 1⋅97; heterogeneity *P* < 0⋅001; *I*^2^ 57⋅2 %) and the MetS components. High triacylglycerols (OR = 1⋅96; 95 % CI 1⋅65, 2⋅32; heterogeneity *P* < 0⋅001; *I*^2^ 82⋅8 %) and high glucose levels (OR = 1⋅60; 95 % CI 1⋅40, 1⋅82; heterogeneity *P* < 0⋅001; *I*^2^ 77⋅8 %) were the components most strongly associated with high ferritin^(20)^. Nevertheless, it is important to note that MetS is characterised by chronic low-grade inflammation^(21)^, which may decrease Fe status. Therefore, we hypothesise that MetS may be positively associated with the different stages of Fe deficiency (ID and IDA) and anaemia. Based on this hypothesis, the objective of the present study was to assess the associations of ID, IDA and anaemia with MetS in Ecuadorian women.

## Materials and methods

### Data source and sampling

This cross-sectional study was based on data from the 2012 Ecuadorian National Health and Nutrition Survey (ENSANUT-ECU, acronym in Spanish). The survey applied a probabilistic, multistage, population-based sampling design, stratified by clusters (household segments) that included 19 706 households to obtain national and sub-regional representativeness (16 sub-regions). The data collection was performed by trained field workers using standardised procedures, protocols and equipment. Besides, the quality and validity of data were controlled at different levels. Detailed information regarding the data collection procedures, approved by the Ethics Committee of the Universidad San Francisco de Quito is available in the official survey book^(22)^. The ENSANUT-ECU databases analysed in this study are publicly available^(23)^. The sample for the current analysis comprised 5894 women aged 20–59 years. The inclusion criterion was complete data of variables of interest: age, sex, weight, height, Hb, serum ferritin, waist circumference (WC), High Density Lipoproteins (HDL), Triacylglyceride (TAG), blood pressure, fasting glucose and C-reactive protein (CRP). Furthermore, inflammation status (CRP > 10 mg/l) was the exclusion criterion^(24)^.

### Assessment of iron deficiency, iron-deficiency anaemia, anaemia and MetS

In the ENSANUT-ECU 2012, the methods used for the biochemical assessment were sodium lauryl sulphate spectrophotometry (Hb), chemiluminescent immunoassay (ferritin), colourimetric enzymatic-assay (HDL, TAG and glucose) and automated nephelometry (CRP). In the survey, the anthropometric and blood pressure measurements followed established protocols. Weight was measured to the nearest 0⋅1 kg using a digital weighing scale. Height and waist circumference were measured to the nearest 0⋅1 cm with a portable stadiometer and an ergonomic circumference measuring tape, respectively. Blood pressure was measured to the nearest 0⋅5 mmHg with a digital tensiometer. All anthropometric variables and blood pressure were collected twice, with an interval of 5 min. When there was a difference of ±0⋅5 kg in weight, or ±0⋅5 cm in height and waist circumference, or ±5 mmHg in blood pressure, a third measurement was made. The mean value was calculated from the two closest values^(22)^.

ID was defined as serum ferritin <15 μg/l^(25)^. Anaemia was defined as Hb < 12 g/dl, after adjustment by altitude^(26)^. IDA was considered as the coexistence of serum ferritin <15 μg/l and anaemia^(24)^. The diagnosis of MetS was based on the harmonised definition (2009)^(9)^, which requires the presence of at least three of the following criteria: WC ≥ 80 cm; fasting glucose ≥ 100 mg/dl; TAG ≥ 150 mg/dl; HDL <50 mg/dl; systolic blood pressure ≥ 130 and/or diastolic blood pressure ≥ 85 mmHg or antihypertensive drug treatment in a patient with a history of hypertension.

### Statistical analysis

The socio-demographic characteristics of the sample were described with frequencies and percentages. Anthropometric variables as height, weight and BMI were described with mean and standard deviation (sd). The remaining continuous variables were described according to their distribution, evaluated by a Kolmogorov–Smirnov test. The prevalence of the stages of Fe deficiency, anaemia and MetS is expressed as frequency, percentage and standard error (se). Prior to the association analysis, the sample was stratified by age decades. The *χ*^2^ test and Fisher's exact test were used to evaluate the possible associations of the stages of Fe deficiency and anaemia with MetS. The prevalence ratio (PR) of ID, IDA and anaemia was estimated considering women without MetS as a reference. The SPSS statistical software package PASW Statistics for Windows version 18.0 (SPSS Inc, Chicago, IL, USA) was used to conduct the analyses.

### Ethical considerations

The current research was conducted under the principles of the Helsinki Declaration^(27)^. In Ecuador, the official public data, as the databases analysed in this study^(23)^, are free access according to the Organic Code of Social Economy of Knowledge, Creativity and Innovation^(28)^. Since this is a secondary analysis of anonymised data from a national survey, which were collected following ethical standards^(22)^, no authorisation is required from a Health Research Ethics Committee.

## Results

Most of the participants (93⋅3 %) were 20–49 years, 16 % self-identified as members of an ethnic minority and 44⋅8 % were in the economic status quintiles Q1 or Q2. Table 1 shows other socio-demographic characteristics of the sample. Mean BMI was 27⋅3 (sd 4⋅7; see Table 2), and 67⋅3 % of the women had excess weight (see Table 1). The proportion of women affected by the MetS components was elevated WC 76⋅5 % (se 0⋅6), reduced HDL 68⋅5 % (se 0⋅6), elevated TAG 26⋅9 % (se 0⋅6), elevated blood pressure 15⋅5 % (se 0⋅5) and elevated fasting glucose 14⋅4 % (se 0⋅5). The total prevalence of MetS was 32⋅3 % (se 0⋅6; Table 3). Overall, 81⋅7 % (se 0⋅5) of the women were not affected by the stages of Fe deficiency or anaemia, and 7⋅1 % (se 0⋅3) had IDA (Table 3).

**Table 1.** Socio-demographic characteristics of participants: Ecuadorian women aged 20–59 years (data from the ENSANUT-ECU 2012)

	*n*^a^	%
Age (years)		
20–29	1752	29⋅7
30–39	2102	35⋅7
40–49	1642	27⋅9
50–59	398	6⋅8
Ethnicity		
Indigenous	569	9⋅7
Afro-Ecuadorian	203	3⋅4
Montubio	173	2⋅9
Mestizo, white and others	4949	84⋅0
Economic status index^b^		
Q1 (poorest)	1300	22⋅1
Q2	1339	22⋅7
Q3	1173	19⋅9
Q4	1091	18⋅5
Q5 (wealthiest)	988	16⋅8
BMI category		
Thinness	72	1⋅2
Normal	1856	31⋅5
Overweight	2441	41⋅4
Obesity	1525	25⋅9

BMI categories were defined according to WHO: thinness (BMI < 18⋅5 kg/m^2^), normal (BMI ≥ 18⋅5–24⋅9 kg/m^2^), overweight (BMI ≥ 25–29⋅9 kg/m^2^) and obesity (BMI ≥ 30 kg/m^2^).

a Total = 5894.

b Women with no data *n* = 3.

**Table 2.** Anthropometrical, biochemical and clinical biomarkers of participants: Ecuadorian women aged 20–59 years (data from the ENSANUT-ECU 2012)

	Mean	sd	Minimum	Maximum
Height (cm)	151⋅9	6⋅1	99⋅9	198⋅3
Weight (kg)	63⋅2	11⋅8	33⋅0	138⋅7
BMI (kg/m^2^)	27⋅3	4⋅7	15⋅0	52⋅7
	Median	IQR	p5	p95
Haemoglobin (g/dl)	13⋅2	1⋅3	11⋅1	14⋅6
Serum ferritin (μg/l)	44⋅0	52⋅0	7⋅0	169⋅3
CRP (mg/l)	1⋅9	1⋅5	1⋅9	7⋅0
Fasting glucose (mg/dl)	88⋅0	13⋅0	72⋅0	109⋅0
HDL (mg/dl)	44⋅0	15⋅0	28⋅0	67⋅0
TAG (mg/dl)	107⋅0	80⋅0	48⋅0	274⋅0
WC (cm)	87⋅2	14⋅4	71⋅2	106⋅7
SBP (mmHg)	113⋅0	15⋅5	96⋅0	138⋅0
DBP (mmHg)	70⋅5	12⋅5	58⋅0	88⋅0

sd, standard deviation; IQR, interquartile range; CRP, C-reactive protein; WC, waist circumference, SBP, systolic blood pressure; DBP, diastolic blood pressure.

For all variables, the significance of the Kolmogorov–Smirnov test was *P* < 0⋅01.

**Table 3.** Prevalence of the stages of iron deficiency and anaemia according to metabolic syndrome diagnosis in Ecuadorian women aged 20–59 years (data from the ENSANUT-ECU 2012)

Deficiency category	Metabolic syndrome								
	Yes			No			Total		
	*n*	%	se	*n*	%	se	*n*	%	se
No deficit	1639	27⋅8	0⋅6	3174	53⋅9	0⋅6	4813	81⋅7	0⋅5
ID	69	1⋅2	0⋅1	299	5⋅1	0⋅3	368	6⋅2	0⋅3
IDA	118	2⋅0	0⋅2	300	5⋅1	0⋅3	418	7⋅1	0⋅3
Anaemia	79	1⋅3	0⋅1	216	3⋅7	0⋅2	295	5⋅0	0⋅3
Total	1905	32⋅3	0⋅6	3989	67⋅7	0⋅6	5894	100⋅00	

se, standard error; ID, Fe deficiency; IDA, Fe-deficiency anaemia.

ID was defined as serum ferritin <15 μg/l as proposed by WHO^(25)^.

Anaemia was defined as Hb <12 g/dl after adjustment by altitude as proposed by WHO^(26)^.

IDA was defined as the presence of serum ferritin <15 μg/l and anaemia^(24)^.

MetS definition was based on the harmonised definition (2009), with at least three of the following criteria: waist circumference ≥ 80 cm; fasting glucose ≥ 100 mg/dl; TAG ≥ 150 mg/dl; HDL < 50 mg/dl; systolic blood pressure ≥ 130 and/or diastolic blood pressure ≥ 85 mmHg or antihypertensive drug treatment in a patient with a history of hypertension^(9)^.

Table 4 reports PR of the stages of Fe deficiency and anaemia (crude and stratified by age) in women with and without MetS. In women less than 50 years, MetS was associated with ID but not with IDA or anaemia. Women of reproductive age (20–49 years) with MetS had a lower prevalence of ID than their counterparts without MetS. PR of ID for these women was (PR (95 % CI; *P*-value)): 0⋅17 (0⋅06, 0⋅46; *P* < 0⋅001) (20–29 years); 0⋅69 (0⋅48, 0⋅99; *P* = 0⋅044) (30–39 years) and 0⋅44 (0⋅29, 0⋅67; *P* < 0⋅001) (40–49 years). On the other hand, women aged 50–59 years with MetS had a lower prevalence of IDA and anaemia than those without MetS (PR (95 % CI; *P*-value)): 0⋅12 (0⋅02, 0⋅96; *P* = 0⋅026) and 0⋅22 (0⋅07, 0⋅64; *P* = 0⋅002), respectively. No significant association was observed between MetS and ID in women aged 50–59 years (*P* = 0⋅660).

**Table 4.** Association of metabolic syndrome with the stages of iron deficiency and anaemia by age, in Ecuadorian women aged 20–59 years (data from the ENSANUT-ECU 2012)

Age	MetS	ID			IDA			Anaemia		
		PR	95 % CI	*P*-value	PR	95 % CI	*P*-value	PR	95 % CI	*P*-value
20–29 years (*n* = 1752)	No	1⋅00	Ref	–	1⋅00	Ref	–	1⋅00	Ref	–
	Yes	0⋅17	0⋅06, 0⋅46	0⋅000	0⋅57	0⋅31, 1⋅06	0⋅067	0⋅66	0⋅36, 1⋅23	0⋅185
30–39 years (*n* = 2102)	No	1⋅00	Ref	–	1⋅00	Ref	–	1⋅00	Ref	–
	Yes	0⋅69	0⋅48, 0⋅99	0⋅044	0⋅85	0⋅61, 1⋅19	0⋅333	0⋅82	0⋅53, 1⋅28	0⋅389
40–49 years (*n* = 1642)	No	1⋅00	Ref	–	1⋅00	Ref	–	1⋅00	Ref	–
	Yes	0⋅44	0⋅29, 0⋅67	0⋅000	0⋅74	0⋅55, 1⋅01	0⋅055	0⋅74	0⋅50, 1⋅10	0⋅138
50–59 years (*n* = 398)	No	1⋅00	Ref	–	1⋅00	Ref	–	1⋅00	Ref	–
	Yes	0⋅54	0⋅09, 3⋅21	0⋅660*	0⋅12	0⋅02, 0⋅96	0⋅026*	0⋅22	0⋅07, 0⋅64	0⋅002

MetS, metabolic syndrome; ID, Fe deficiency; IDA, Fe-deficiency anaemia; PR, crude prevalence ratio; Ref, reference category; 95 % CI, 95% confidence interval.

* Two-tailed *P*-values for Fisher's exact test, and the remaining two-tailed *P*-values were assessed by *χ*^2^ test.

## Discussion

This work was based on the hypothesis that MetS may be positively associated with the different stages of Fe deficiency (ID and IDA) and anaemia, but the results show the opposite. In Ecuadorian women of reproductive age (20–49 years), MetS is associated with a lower prevalence of ID. Additionally, in women aged 50–59 years, MetS is associated with a lower prevalence of IDA and anaemia. The discrepancies between these two age groups could be attributed to their physiological differences; however, it must be emphasised that the proportion of postmenopausal women is 6⋅8 % of the sample.

Although we did not identify reports evaluating the association between MetS as an independent variable and the different stages of Fe deficiency as dependent variables, in Latin America, this association has been studied in the opposite direction. Leiva *et al.* found that, in Chilean adults aged 45–65 years, subjects with higher serum ferritin levels had a greater risk of MetS (OR = 3⋅36; 95 % CI 1⋅14, 4⋅20; *P* < 0⋅001)^(29)^. Moreover, they identified that women with MetS had higher levels of Fe parameters than those without MetS: serum Fe (mean (sd) = 122⋅9 (54⋅4) μg/dl *v.* 108⋅8 (43⋅1) μg/dl; *P* < 0⋅03), serum ferritin (geometric mean (range) = 53⋅9 (34⋅1, 84⋅8) μg/l *v.* 27⋅4 (12⋅6, 59⋅5) μg/l; *P* < 0⋅001) and total body Fe (mean (sd) = 6⋅3 (2⋅3) mg/kg *v.* 4⋅1 (2⋅8) mg/kg; *P* < 0⋅001)^(29)^. Similarly, in the present paper, higher serum ferritin levels were identified in women with MetS compared with those without MetS (median (interquartile range) = 55⋅0 (66⋅0) μg/l *v.* 40⋅0 (46⋅0) μg/l; *P* < 0⋅001) and MetS was associated with a higher prevalence of Fe excess (serum ferritin >150 μg/l^(24)^; PR = 2⋅49; 95 % CI 2⋅05, 3⋅02; *P* < 0⋅001); results not reported. In this context, we also highlight a study conducted in Brazilian adults reporting associations of high haem Fe intake with increased risk of MetS (OR = 2⋅39; 95 % CI 1⋅10, 5⋅2) and hypertriglyceridaemia (OR = 2⋅51; 95 % CI 1⋅06, 5⋅91)^(30)^.

The causal direction of the association between MetS and Fe nutritional status is unclear. Despite this fact, from biological plausibility, some hypotheses that could explain this association are as follows: the dietary intake of haem Fe increases body Fe stores, which predict the development of type 2 diabetes mellitus^(31)^; according to animal model studies, Fe overload causes pancreatic β-cells dysfunction, associated with oxidative stress^(32)^ and mitochondrial dysfunction^(33)^; elevated Fe levels are associated with adipocyte insulin resistance and decreased plasma adiponectin^(34)^; and Fe overload increases muscle glucose uptake despite reduced insulin signalling in animal models^(35)^. Finally, elevated serum ferritin does not necessarily reflect high Fe stores^(36)^. The levels of this protein are also associated with the chronic low-grade inflammation in MetS^(37)^. Furthermore, in men and postmenopausal women, MetS is associated with lower serum ferritin levels than those established by WHO as cut-off points for Fe overload^(38)^. It is important to note that the median of serum ferritin (42 μg/l (IQR 48⋅0)) observed in Ecuadorian women (20–49 years) seems to be higher than the median values reported from adult women in developed countries, which ranged from 32 μg/l in women aged 20 to <24 years to 41 μg/l in women aged 48 to <52 years^(24)^. This data could be considered as a success of Fe fortification strategies in Ecuador. However, based on the possible relationship between MetS and Fe status, a detailed follow-up of iron fortification programmes should be encouraged.

On the other hand, according to the new perspectives of nutrition science, to explore the effects of food on diet-related NCDs, it is necessary to move from the nutrient-focused approach to an analysis of the dietary patterns of the population^(2)^. This new paradigm contrasts with the hegemonic approaches in food and nutrition policies^(2)^, which have been useful in reducing micronutrient deficiencies^(39)^.

It is important to take into account that in Latin American countries, the major determinant of the increase in overweight, obesity and diet-related NCDs is a diet high in saturated fat, sugar and sodium, with high consumption of food of animal origin, refined cereals, and processed and ultra-processed products^(40)^. This diet is replacing the traditional diet and, therefore, the purchase and consumption of natural and minimally processed foods^(41,42)^, which affects food sovereignty^(8)^ and planetary health^(43)^. All this, in the context of the transformation of the food system into one controlled by international agribusiness, food industries, food retailers and food service companies^(40)^. Thus, greater attention is needed not only to dietary patterns but also to the food system that promotes the consumption of these patterns.

In addition, the mandatorily fortified foods in the region are wheat flour in eighteen countries, maize flour in five countries and milled rice in three countries^(44)^. These cereals are widely consumed^(45,46)^, but contain little fibre and protein^(47)^ and high glycaemic index^(48)^. Diets high in glycaemic index may elevate the risk of type-2 diabetes^(49,50)^ and other cardiometabolic diseases^(50)^. In the region, other mandatorily fortified vehicles with low nutritional quality are vitamin A-fortified sugar in five countries and iodine-fortified salt in eighteen countries^(44)^.

This is relevant in the context of DBM, as observed in Ecuadorian women with the coexistence of MetS (32⋅3 %) and IDA (7⋅1 %) at the population level. DBM requires policies that simultaneously and synergistically address undernutrition, overweight, obesity and diet-related NCDs^(1)^.

An alternative to face all forms of malnutrition is to implement public policies that promote a food system that ensures healthy and sustainable food for the entire population. Such policies should address nutrition from a dietary pattern approach, integrating the cultural, social, environmental, health^(51)^, nutritional and political dimensions of dietary habits. Examples of this are the Brazilian^(52)^ and Uruguayan^(53)^ dietary guidelines that recommend a diet based on natural or minimally processed foods and home-cooked meals with a wide variety of foods of plant origin and moderate amounts of foods of animal origin, limiting the use of culinary ingredients and processed foods, avoiding ultra-processed products, and eating in company and attentively to enjoy food.

Currently, the ENSANUT ECU reported that, in adults, the mean calorie intake is 1822 kcal (women) and 2143 kcal (men), the contribution of each macronutrient to the total energy intake is 61 % for carbohydrate, 13 % for protein and 26 % for fat (12 % for saturated fat)^(22)^. However, the description of the dietary pattern of this population is not reported.

Moreover, in Ecuador, rice is the main contributor to the daily intake of non-haem Fe and total Fe, providing 24⋅2 and 19⋅4 %, respectively^(22)^. The above data is interesting because, in the country, the food chosen for mandatory fortification with Fe and other micronutrients is wheat flour^(54)^, while rice fortification is a voluntary measure^(41)^, for which no reports or efficacy assessments were found. This situation requires close monitoring, since voluntary fortification initiatives, along with mandatory fortification policies and supplementation programmes (generally targeting children and women of reproductive age), could lead to an excess of micronutrients with negative health consequences^(55)^. For this reason, it is required to control voluntary fortification and excessive intake of micronutrients from non-food sources.

Regarding the strengths of the study, it is highlighted that it was conducted on a sample of adult women derived from ENSANUT-ECU 2012, which provides information on the health and nutritional status of the population to contribute to the design of public policies and programmes^(22)^. Besides, it is an initial approach in the exploration of possible interactions between overnutrition and undernutrition in Ecuadorian women and is relevant for the generation of hypotheses that could be evaluated in further longitudinal studies. Finally, we recognise that the present work presents some limitations: first, the cross-sectional design does not allow causality analysis; second, daily Fe intake, which could mediate in the relationship between Fe nutritional status and MetS, was not assessed. Intake of over-the-counter vitamin C and minerals should also be evaluated since they could play an important role in the present and future health of the Ecuadorian population.

## Conclusions

The present study showed that, in Ecuadorian women of reproductive age, MetS is associated with a lower prevalence of Fe deficiency without anaemia. Additionally, the coexistence of MetS and Fe-deficiency anaemia was identified at the population level. These findings require an analysis that transcends the nutrient-focused approach and considers the dietary patterns of the population, as well as the food system that promotes them. This could provide key elements for developing public policies that simultaneously address all forms of malnutrition while promoting food sovereignty.
